# Single session of laser photobiomodulation for symptom management of oral lichen planus: a retrospective study

**DOI:** 10.1007/s10103-023-03706-4

**Published:** 2023-01-19

**Authors:** Andrea Roccon, Francesco Cavallin, Gastone Zanette, Christian Bacci

**Affiliations:** 1 Department of Neurosciences, Section of Clinical Dentistry, University of Padua, via Giustiniani, 2, Padova, Italy; 2 Independent Statistician, Solagna, Italy

**Keywords:** Laser, Oral lichen planus, Photobiomodulation, LLLT, Flat top handpiece, VAS

## Abstract

This study aimed to evaluate the effectiveness of a single session of laser photobiomodulation (PBM) with flat top handpiece in reducing painful symptoms in patients with Oral Lichen Planus (OLP). The clinical charts of 20 patients of the Dental Clinic of University of Padua (Italy) who underwent a single laser PBM to manage OLP symptomatology were retrospectively analyzed. A 980 nm diode laser and a flat top handpiece with a 1-cm^2^ spot area were employed to perform the PBM with a single session protocol. VAS pain scores were assessed before and after the laser PBM, the day after, and on the 7th and 30th days after the treatment. No adverse effects occurred within 30 days after treatment. The mean VAS-pain score was 3.8 (SD 2.3) before PBM; 2.6 (SD 2.0) after PBM; 1.9 (SD 2.2) on day 1; 2.0 (SD 2.3) on day 7; and 1.5 (SD 2.2) on day 30. VAS pain decreased significantly over time (*p* < 0.0001). A single session of laser PBM may be safe and effective in reducing pain for symptomatic OLP patients. Further investigations are required to include placebo or topical corticosteroids as comparators.

## Introduction

Oral lichen planus (OLP) is a chronic non-infective inflammatory disease that affects oral mucosa [1–3]. This disease has a worldwide prevalence around 1% [4] and mainly affects adults aged 30 to 60 with a predilection for women [5].

**Fig. 1:**
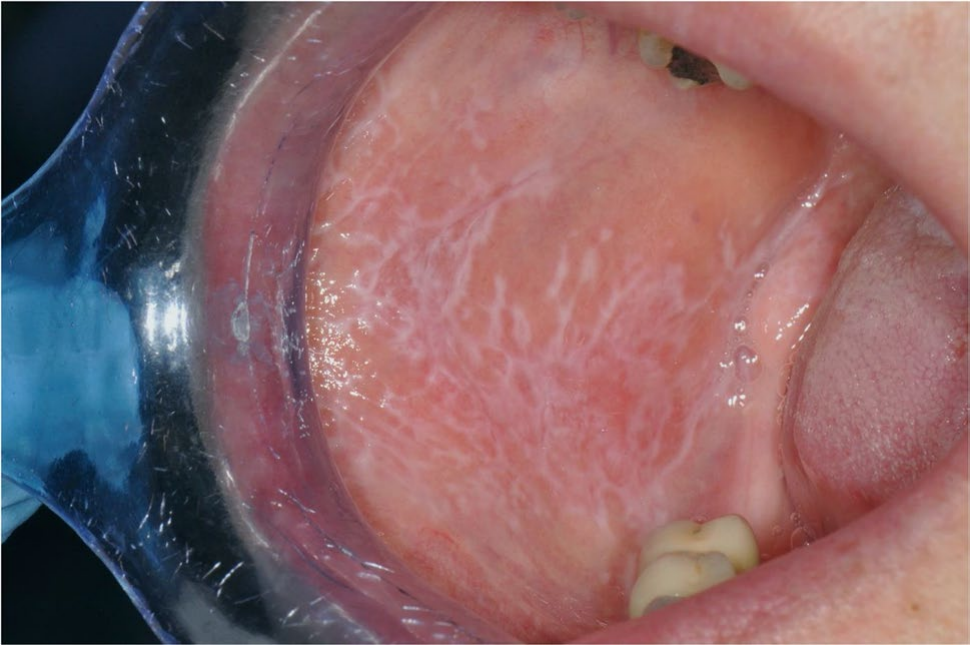
Reticular oral lichen planus affecting the buccal mucosa (Thongprasom score 1)

**Fig. 2:**
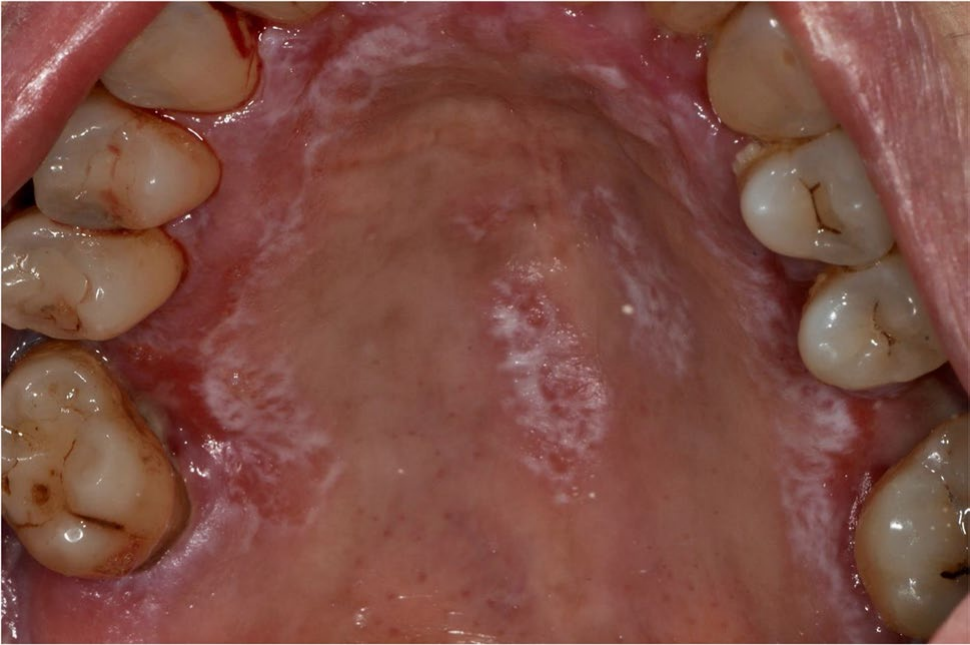
Erosive oral lichen planus affecting the palate (Thongprasom score 5)

OLP is generally classified into reticular and erosive form [3]. Reticular OLP is the most frequent subtype, is generally asymptomatic, and often affects buccal mucosa bilaterally (67.5% of cases) with white and arborescent striae (the so-called Whickam Striae) [4] (Fig. 1). Erosive OLP is less common, is often symptomatic, and displays atrophic, erythematous areas, and ulcerations of various magnitude surrounded by white striae [3] (Fig. 2). Besides this classification, Thongprasom et al. proposed a scale to determine the severity of OLP, which is one of the most widely used in the literature and consists of a 0 to 5 score: “ 0 = healthy mucosa; 1 = mild white striae without erythematous area; 2 = white striae with an atrophic area less than 1 cm^2^; 3 = white striae with an atrophic area more than 1 cm^2^; 4 = white striae with a erosive area less than 1cm^2^; 5 = white striae with a erosive area more than 1cm^2^” [6].

There are controversies about the exact etiology and pathogenesis of OLP [7–9]. An important role in the pathogenesis of OLP is attributed to an immune dysregulation that involves cell-mediated immunity and causes a damage to epithelial keratinocytes [10]. Indeed, the inflammatory infiltrate in OLP mainly consists of T cells and macrophages [8]. Peculiar findings of the histopathology of oral lichen planus are the liquefaction of the basal cells with the formation of Civatte bodies (apoptotic keratinocytes) and the presence of a band-like lymphocytic infiltrate at the interface between epithelium and lamina propria [3, 11–13]. Although OLP manifestations may be clear on the oral mucosa, the clinical diagnosis, as with all the lesions in oral pathology, needs to be confirmed by histopathological examination [14–16]. The biopsy can also allow excluding the presence of dysplasia, which is a fundamental parameter for the prognosis and treatment of the patient [17, 18].

The therapy is required only in case of symptomatic OLP [3, 17]. The first line treatment is the topical application of corticosteroids [6, 19–21], and the therapy generally lasts 1 − 2 months with 2 − 3 daily topical application. However, long-term corticosteroid therapy can lead to several side effects; hence, alternative treatments have been proposed [22]. Among these, laser photobiomodulation (PBM) can be a valid treatment option for OLP which does not require any medication and has less side effects than corticosteroids [23, 24]. Laser PBM, formerly defined as low level laser therapy (LLLT), is a medical treatment that uses a coherent beam of light that interacts with specific substances in the tissues, called chromophores, to obtain effects in terms of analgesia, anti-inflammatory, and biostimulating effect [25–27].

This study aimed to evaluate the effectiveness of a single session of laser photobiomodulation with diode laser in reducing painful symptoms in patients with OLP.

## Materials and methods

This is a retrospective study including all 20 consecutive patients who were treated with a single session laser PBM to manage OLP symptomatology at the Dental Clinic of University of Padua from January to June 2021. Inclusion criteria were as follows: (1) patients followed for OLP in the Oral Pathology Unit; (2) clinical and histopathological diagnosis of OLP according to Van Der Meij 2003 [13]; (3) age > 18 years; and (4) presence of symptomatic lesions (visual analogue scale pain > 0) treated with a single session laser PBM. Some patients were previously treated with other therapies in the past. Simultaneous or recent treatment with corticosteroids, immunomodulatory, or antifungal drugs were considered exclusion criteria. All patients signed an informed consent for PBM.

The following data were collected from the clinical charts: age, sex, OLP lesion localization, Thongprasom score [20], treatment-related adverse effects, and visual analogue scale for pain (VAS pain) score.

A 980-nm diode laser (Doctor-Smile® Wiser L A 8D0 001.3) and a flat top handpiece with a 1-cm^2^ spot area (Doctor-Smile® AB2799) were employed to perform a single session of laser PBM. Laser energy was delivered with a spot-technique in non-contact mode, with a variable number of spots to cover all the size of the lesion and the area over the border for 5 mm. Some patients presented symptomatic and asymptomatic lesions, and both were treated. To ensure a fluence of 10 J/cm^2^, the diode laser was set as follows: output power of 0.5 W with continuous wave (power density = 0,5 W/cm^2^) and time of application 20 s per point.

VAS pain scores were assessed before and after the laser PBM, the day after, and on the 7th and 30th days after the treatment. The patient recorded the VAS pain score by using a score collection form and communicated the data by telephone due to COVID-19 restrictions. If VAS pain score was ≥ 4 before PBM, a therapy based on corticosteroids and antifungals (fluocinonide and miconazole: 3 topical application per day for 4 weeks) was also prescribed. In case of persistence of symptoms, patients could take this medication and record the VAS pain score before starting the therapy. These patients were considered as dropouts.

The statistical analysis was carried out with R 4.1 (R Foundation for Statistical Computing, Vienna, Austria [28]). We estimated that 20 patients were required to have an 80% chance of detecting, as significant at the 5% level, a standardized effect sized of 0.66 in the change of VAS pain scores. Continuous data were reported as mean and standard deviation (SD), and categorical data as frequency and percentage. Longitudinal data of VAS pain scores were analyzed using mixed regression models. In addition, VAS pain scores at each postoperative time were compared to baseline scores (before PBM) with a paired-sample Student’s *t* test with Benjamini–Hochberg correction for multiple tests (adjusted *p* values ​​were indicated with *p*_adj_). The association between the change of VAS pain scores over time and some clinically relevant parameters (age, sex, number of lesion spots, and Thongprasom score) was investigated with additional mixed regression models including time and one parameter in each model (due to the limited sample size). As two subjects dropped out the follow-up before 15-day and 30-day assessments, a sensitivity analysis was performed by reasonably imputing the missing VAS pain score with the baseline VAS pain score (to represent the increase in pain experienced by the subject if the drug was not taken). All tests were 2-sided, and a *p* value of less than 0.05 was considered statistically significant.

## Results

The analysis included 20 patients (12 women and 8 men; mean age 59 years) who were treated with a single session laser PBM to manage OLP symptomatology during the study period. Patient characteristics are reported in Table 1. OLP lesions were found in the buccal mucosa (90%), gingiva (35%), tongue (25%), hard palate (15%), oral floor (5%), and labial mucosa (5%). At the time of the treatment, Thongprasom scores were 1 in seven patients (35%), 2 in one patient (5%), 3 in six patients (30%), 4 in two patients (10%), and 5 in four patients (20%).

**Table 1:** Characteristics of patients who were treated with a single session laser PBM to manage OLP symptomatology

Number of patients	20
Females Males	12 (60%) 8 (40%)
Age, years: mean (SD)	59 (14)
Site of OLP lesions (not mutually exclusive)	
Buccal mucosa Gingiva Tongue Hard palate Oral floor Labial mucosa	18 (90%) 7 (35%) 5 (25%) 3 (15%) 1 (5%) 1 (5%)
Thongprasom score	
1 2 3 4 5	7 (35%) 1 (5%) 6 (30%) 2 (10%) 4 (20%)

*OLP*, oral lichen planus; *PBM*, photobiomodulation; *SD*, standard deviation

No adverse effects occurred after the treatment with laser PBM. Six patients (30%) spontaneously requested further laser PBM sessions in the future. Two patients (10%) dropped out from follow-up at 8 and 15 days after PBM because they started the fluocinonide/miconazole therapy due to lack of pain relief (VAS scores were 7.5 and 4.9, respectively).

VAS pain scores decreased significantly over time (*p* < 0.0001; Table 2), and a statistically significant reduction was recorded from baseline to after PBM (*p*_adj_ = 0.01), day 1 (*p*_adj_ = 0.005), day 7 (*p*_adj_ = 0.003), and day 30 (*p*_adj_ = 0.004). No statistically significant associations were found between VAS over time and age (*p* = 0.61), sex (*p* = 0.37), number of spots (*p* = 0.48), and Thongprasom score (*p* = 0.09).

**Table 2:** VAS pain scores (before and after the laser PBM, the day after, and on the 7th and 30th days after the treatment) in patients who were treated with a single session laser PBM to manage OLP symptomatology

Analysis	Variable	Before PBM (baseline)	After PBM	Day 1	Day 7	Day 30
Main analysis	VAS: mean (SD)	3.8 (2.3)	2.6 (2.0)	1.9 (2.2)	2.0 (2.3)	1.5 (2.2)
Sensitivity analysis	VAS: mean (SD)	3.8 (2.3)	2.6 (2.0)	1.9 (2.2)	2.0 (2.3)	2.0 (2.5)

VAS was measured in centimeters. *OLP*, oral lichen planus; *PBM*, photobiomodulation; *SD*, standard deviation

In the sensitivity analysis, VAS pain scores decreased significantly over time (*p* = 0.002; Table 2), and the reduction was statistically significant after PBM (*p*_adj_ = 0.01), at day 1 (*p*_adj_ = 0.008), day 7 (*p*_adj_ = 0.003), and day 30 (*p*_adj_ = 0.02). Patients with higher Thongprasom score reported higher VAS pain scores over time (*p* = 0.04), while no statistically significant associations were found between VAS over time and age (*p* = 0.36), sex (*p* = 0.33), and number of spot (*p* = 0.44).

## Discussion

Our findings suggest that a single session of laser PBM may provide some advantages in the reduction of pain for symptomatic OLP.

The literature offers several trials and systematic reviews supporting the efficacy of laser PBM in reducing symptoms and clinical signs in OLP [23, 24, 26, 27, 29–34], but those protocols included many PBM sessions (8 to 12) and multiple sessions during the week. For example, Dillenburg et al. found improvement in symptoms, clinical signs, and post-treatment relapse with PBM vs. topical clobetasol for OLP treatment in 42 patients, who underwent a photobiomodulation protocol including 12 sessions, 3 times a week [26]. Further, Jajarm et al. reported comparable improvements in symptoms and clinical signs with PBM vs. dexamethasone rinses, but the protocol included 10 PBM sessions, twice a week [25]. Of note, such protocols require considerable compliance by the patient who is expected to attend the clinics several times for the therapy sessions with the laser. PBM is therefore an indicated treatment for patients with adequate time and means to travel.

Our study investigated the effectiveness of a single laser PBM session during the COVID-19 pandemic, where the choice of a single PBM session was mandatory to reduce visits at the hospital. However, a single laser administration (or any smaller number of sessions) would allow an easier and more suitable treatment to a higher number of patients suffering from OLP.

This study differs from current scientific literature for the use of a flat top handpiece in the treatment of OLP. According to the literature, PBM performed with a flat top handpiece would be more effective, predictable and reproducible [35]. The use of this handpiece and of the “spot technique” application technique allows to accurately calculate the amount of energy delivered to the tissues and to make the protocol easily reproducible. In literature, laser PBM protocols for the treatment of OLP are not always reproducible due to missing information in the description of the PBM laser protocol used. The laser protocol of our study delivered a fluence of 10 J/cm^2^, in accordance with the Clinical Recommendations in Dentistry of the Italian Ministry of Health of 2017 [36]. This value of fluence would allow to obtain an analgesic and anti-inflammatory action on the mucous membranes, two fundamental effects in the management of symptoms of OLP [37]. However, there are no guidelines, Consensus Report, or Position Paper that establish a “Gold Standard” among the PBM protocols in the literature [38].

Although limited to a single session, our findings confirm the data in the literature about the absence of side effects after laser PBM, which is a very relevant data for the treatment of a chronic disease such as OLP [24]. Indeed, commonly used corticosteroid-based therapies can produce, especially if prolonged over time, undesirable effects such as secondary candidiasis, mucosal atrophy, adrenal insufficiency, gastrointestinal problems, hypertension, and diabetes [27].

Our study has some limitations that should be considered by the reader. The retrospective design and the limited sample size suggest caution in the interpretation of the findings. In addition, the absence of a control group or treatment does not allow to exclude any placebo effect in the reduction of painful symptoms [39, 40], which may be a bias in studies assessing subjective outcomes or pain control, and it may explain our results obtained with only a single session of therapy [41]. Further limitations include the absence of a clinical evaluation of OLP by using the Thongprasom score after laser PBM and the follow-up limited to 30 days and a the absence of VAS anxiety scale to evaluate patient’s feelings about the procedure and the perceived pain [42].

## Conclusion

Our findings suggest that a single session of laser PBM may provide some advantages in the reduction of pain for symptomatic OLP, with no undesirable effects. Randomized controlled trials including placebo or topical corticosteroids as comparator would be required to assess the efficacy of the single session laser PBM with a higher level of evidence.
